# The Nurses' Co-Operation

**Published:** 1920-01-31

**Authors:** 


### The Nurses' Co-operation.

THE following statement forms a portion of a much longer document, dated 22 Langham Street, Portland Place, W. 1, January 1920, which has just been issued "dealing with many mis-statements and inaccuracies" "by the leaders in this agitation":

"*From the members of the Nurses' Co-operation to the nurses on the nursing staff.*

"The following figures show the response to the circular sent to you on December 29 last:

| | |
|---|---|
| Answers in the affirmative (that is, upholding the present constitution) ... ... | 400 |
| Answers in the negative ... ... ... | 11 |
| Answers refused or withheld ... ... ... | 17 |
| Nurses who wish to remain neutral ... ... | 2 |
| Letters giving reasons for not signing, etc. | 13 |
| Nurses who have not replied (twenty-four of this number being abroad) ... ... | 50 |
| This makes the total number on the staff | 493 |

"These figures speak for themselves, and in addition a large number of letters has been received from nurses expressing disapproval of the agitation and urging that the leaders of it should be removed from the staff.

"In view of the above overwhelming figures it is clear that the claim made by the leaders in this agitation that they represent the views of the nurses on the staff, is without foundation."

The nurses of the Co-operation have shown great wisdom, courage, and business acumen, which does them credit, and should ensure to them a continuance of the Nurses' Co-operation on sound lines, calculated to protect the best interests of every working nurse amongst them for all time. The members of the Nurses' Co-operation know all the facts which point the way quite clearly to the course which the majority should take without hesitation or delay. Then, having all the force of this the greatest Co-operation of women workers engaged in nursing in the world, by the exercise of sound judgment on the part of its nurse-members, so long as they remain united in their mutual interests, a bright and ever-extending career of usefulness and benefit should continue theirs throughout their working lives.

